# Supplementary material for: Large-scale characterization of drug mechanism of action using proteome-wide thermal shift assays
Source: bioRxiv. 2024 Aug 14:2024.01.26.577428. Originally published 2024 Jan 27. Preprint. [Version 4] doi: 10.1101/2024.01.26.577428 (PMC10849652; doi:10.1101/2024.01.26.577428)
Supplement: Supplement 11 — This document contains the unedited western scans for the panels displayed in Figure 2F and Figure 2 – figure supplement 1D. [file media-11.pdf]

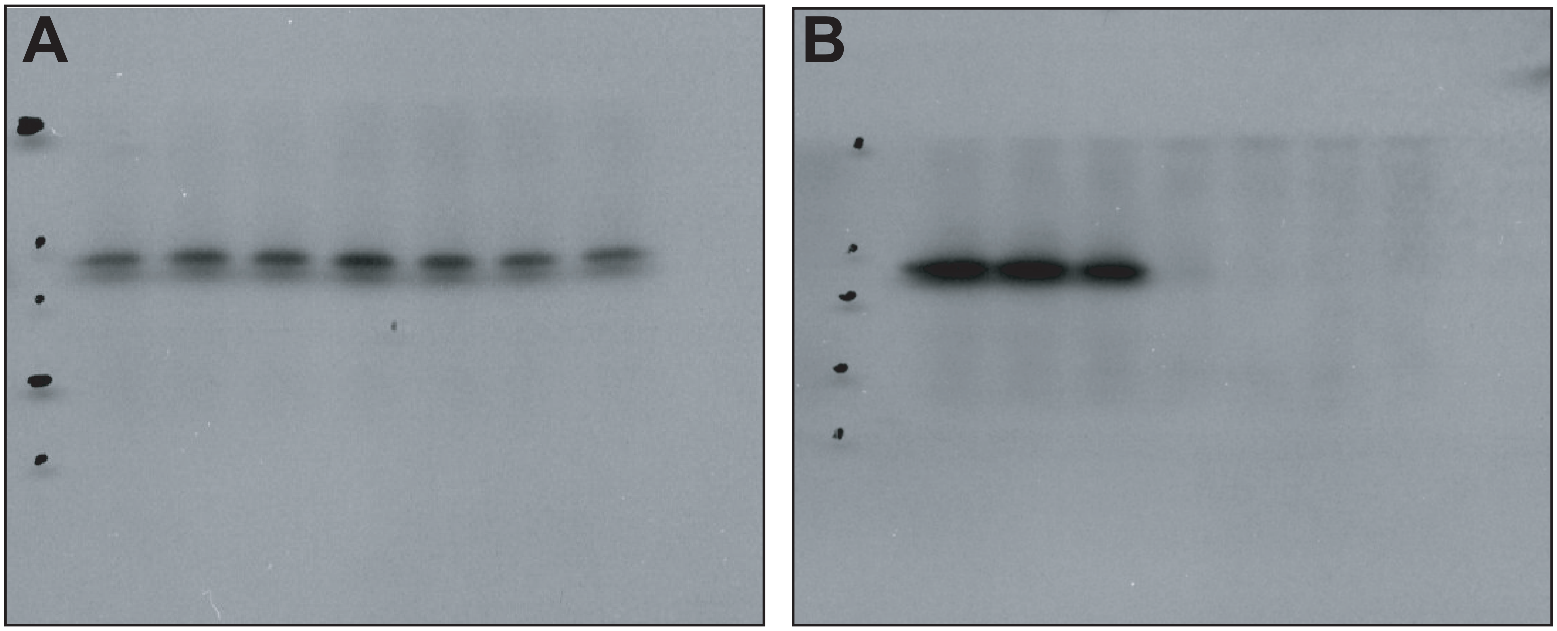

Figure 2F. Unedited scans of TCTP (A) and pTCTP (B).

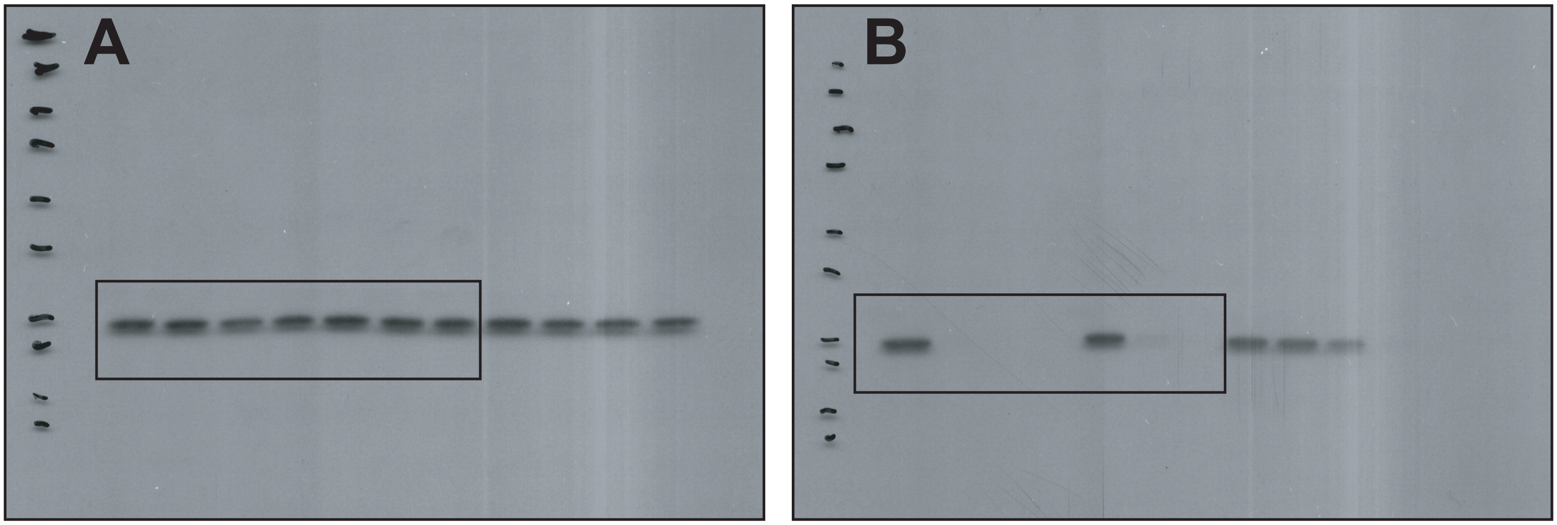

Figure 2 - figure supplement 1D. Unedited scans of TCTP (A) and pTCTP (B).

Figure 2 - source data 3
