# Supplementary figures and images for: Large-scale characterization of drug mechanism of action using proteome-wide thermal shift assays
Source: bioRxiv. 2024 Aug 14:2024.01.26.577428. Originally published 2024 Jan 27. Preprint. [Version 4] doi: 10.1101/2024.01.26.577428 (PMC10849652; doi:10.1101/2024.01.26.577428)

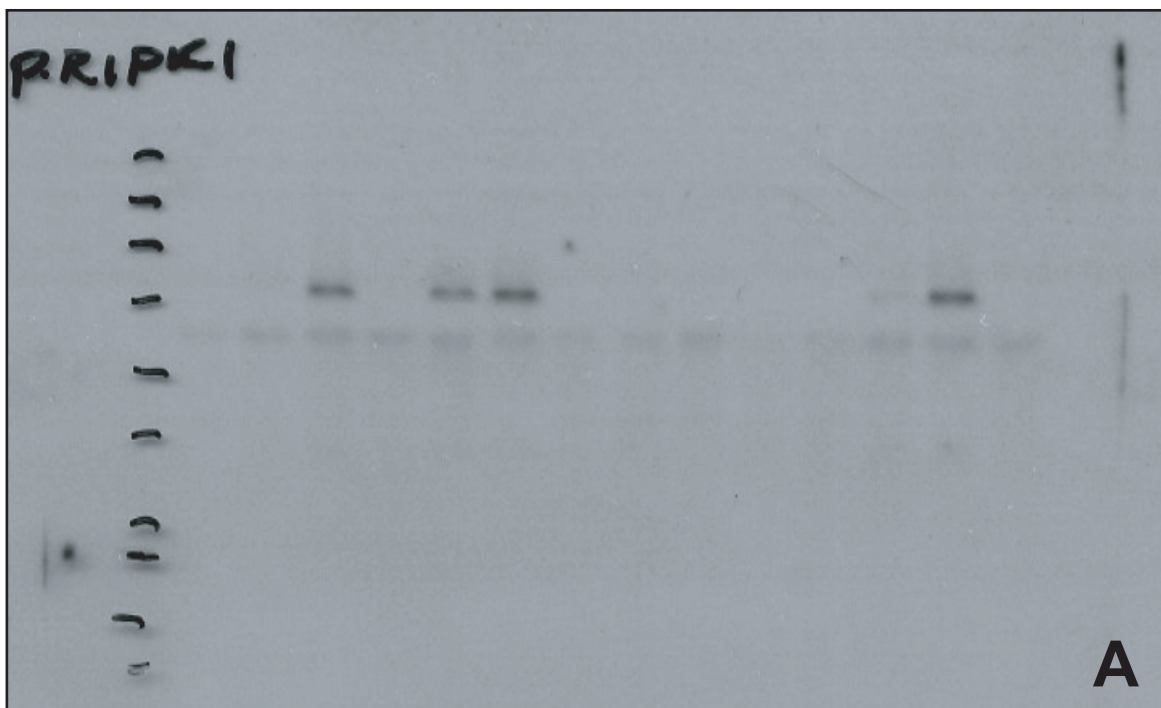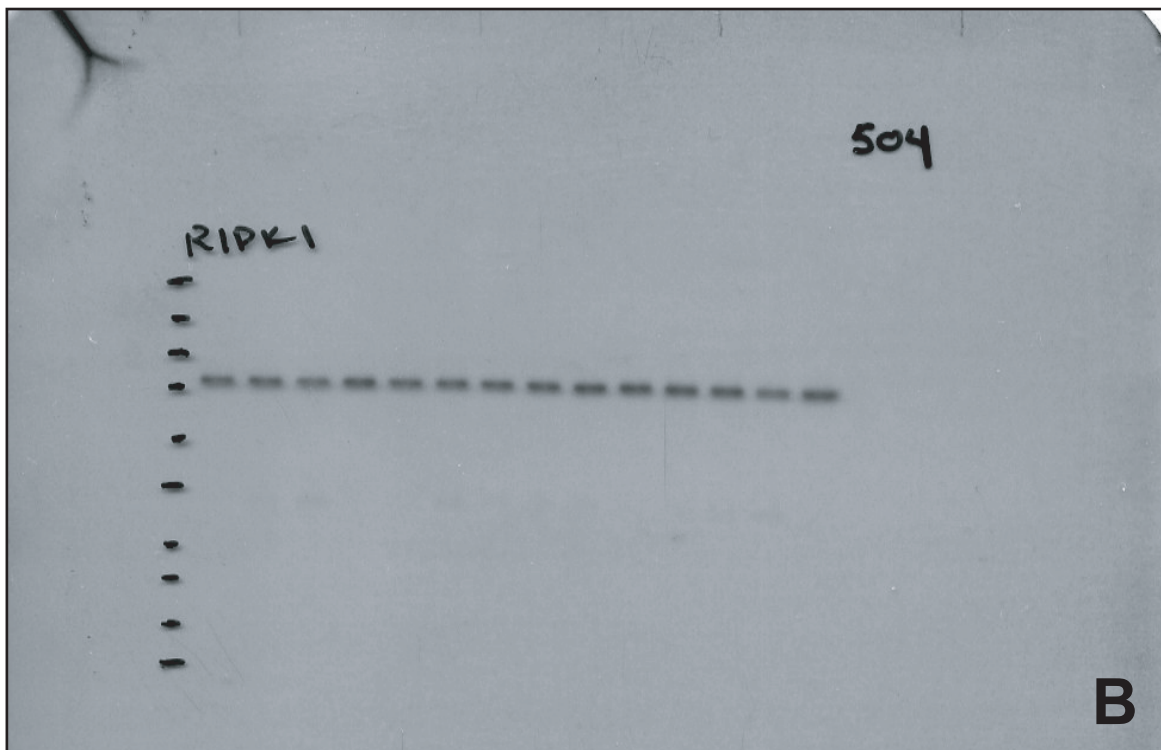

Figure 4G. Unedited scans of pRIPK1 (A) and RIPK1 (B).

Figure 4 - source data 2

Supplement: Supplement 13 — This document contains the unedited western scans for the panel displayed in Figure 4G. [file media-13.pdf]

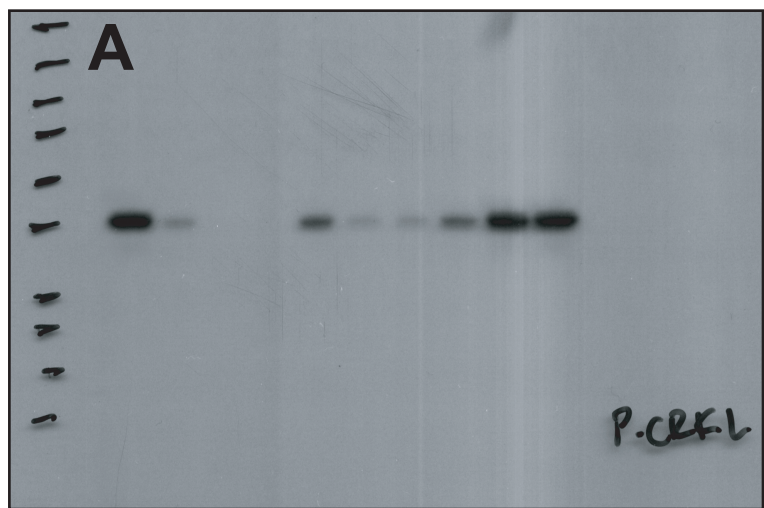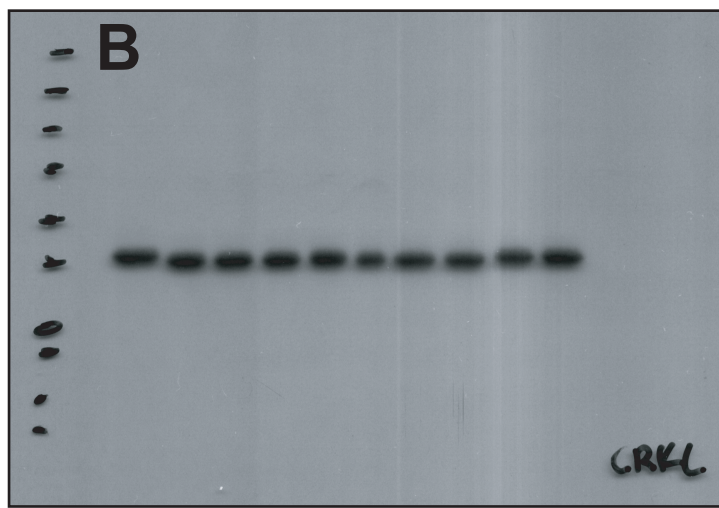

Figure 5C. Unedited scans of pCRKL (A) and CRKL (B).

Figure 5 - source data 1

Supplement: Supplement 14 — This document contains the unedited western scans for the panels displayed in Figure 5C. [file media-14.pdf]
