# Supplementary material for: Large-scale characterization of drug mechanism of action using proteome-wide thermal shift assays
Source: bioRxiv. 2024 Aug 14:2024.01.26.577428. Originally published 2024 Jan 27. Preprint. [Version 4] doi: 10.1101/2024.01.26.577428 (PMC10849652; doi:10.1101/2024.01.26.577428)
Supplement: Supplement 18 [file NIHPP2024.01.26.577428v4-supplement-18.pdf]

bioRxiv preprint doi: https://doi.org/10.1101/2024.01.26.577428; this version posted August 14, 2024. The copyright holder for this preprint (which was not certified by peer review) is the author/funder, who has granted bioRxiv a license to display the preprint in perpetuity. It is made available under aCC-BY 4.0 International license.

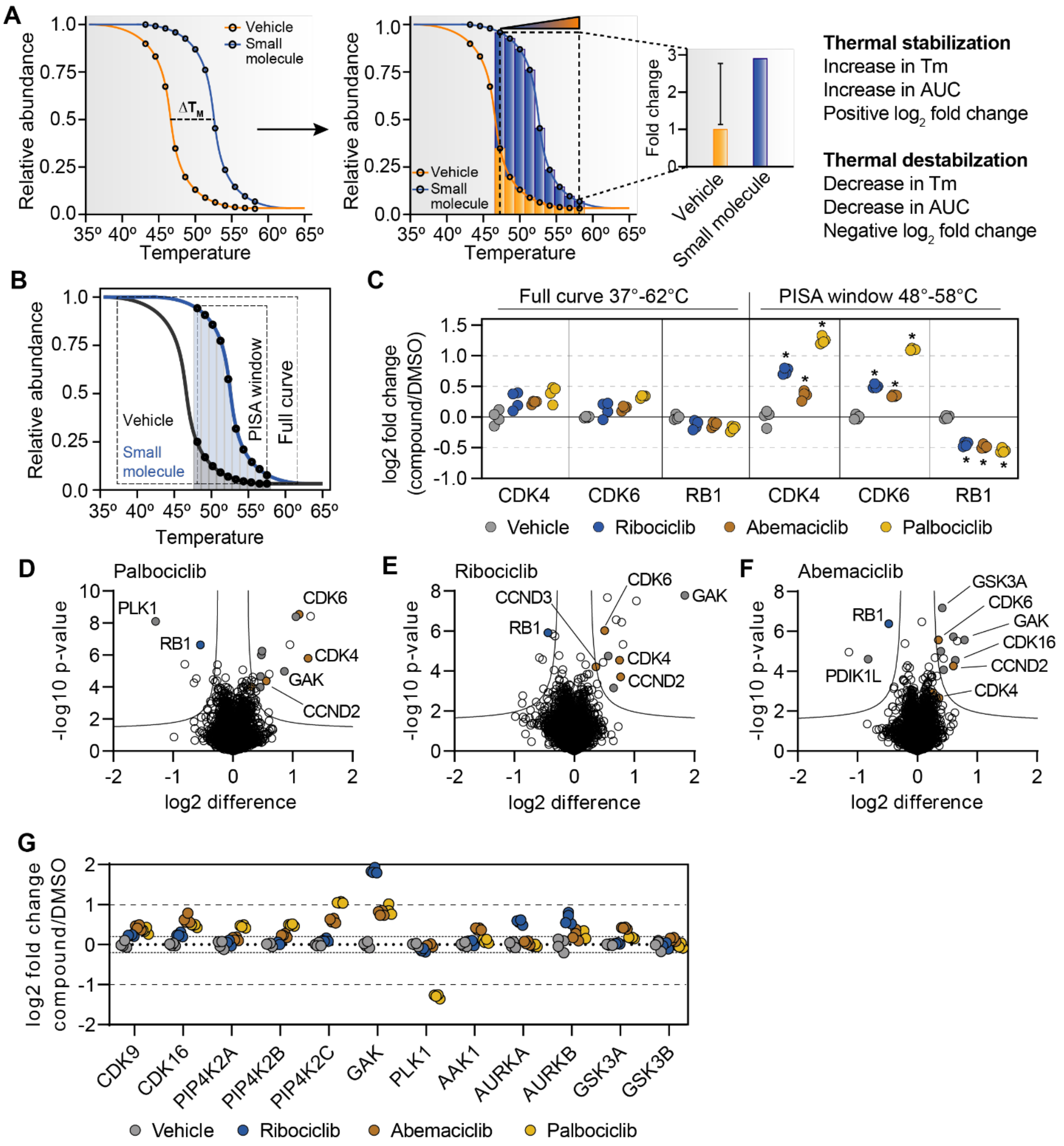

Figure 1 - Figure supplement 1

Figure 1 – figure supplement 1. Establishing a robust and efficient workflow for assessing changes in protein thermal stability in living cells. A. Schematic depicting how thermal shifts are measured in a PISA experiment. An increase in melting temperature (thermal stabilization) will result in an increase in the AUC and a positive log_2 fold change in soluble protein abundance. A decrease in melting temperature (thermal destabilization) will result in a decrease in the AUC and a negative log_2 fold change in soluble protein abundance. B. PISA schematic depicting a thermal window vs. a full melting curve. C-G. K562 cells (N=4) were treated with 10 μM palbociclib

bioRxiv preprint doi: https://doi.org/10.1101/2024.01.26.577428; this version posted August 14, 2024. The copyright holder for this preprint (which was not certified by peer review) is the author/funder, who has granted bioRxiv a license to display the preprint in perpetuity. It is made available under aCC-BY 4.0 International license.

(D), ribociclib (E), or abemaciclib (F) for 15 minutes and assayed using PISA. (C) The $\log_2$ fold changes for specific proteins are plotted for each treatment using a full melting curve (left) or thermal window (48°C-58°C; right). (D-F). Data is presented as a volcano plot to highlight significant changes in abundance utilizing the thermal window (48°C-58°C). Significant changes were determined using a permutation-based FDR (FDR – 0.05, S0 – 0.1). (G). Log_2 fold change values for selected proteins following treatment with each compound in D-F.

bioRxiv preprint doi: https://doi.org/10.1101/2024.01.26.577428; this version posted August 14, 2024. The copyright holder for this preprint (which was not certified by peer review) is the author/funder, who has granted bioRxiv a license to display the preprint in perpetuity. It is made available under aCC-BY 4.0 International license.

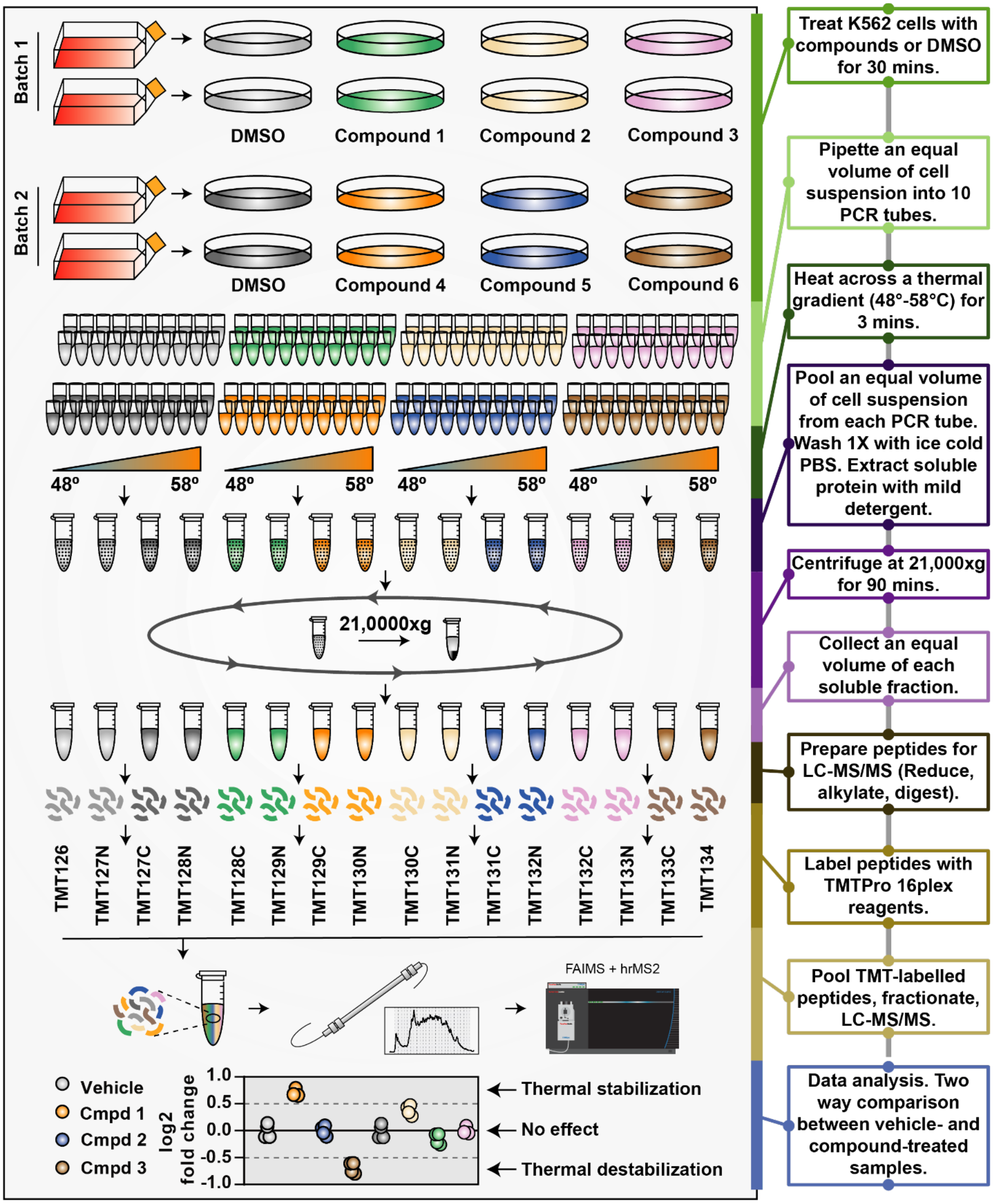

Figure 1 - Figure supplement 2

Figure 1 – figure supplement 2. Establishing a robust and efficient workflow for assessing changes in protein thermal stability in living cells. K562 cells were treated with each of the

bioRxiv preprint doi: https://doi.org/10.1101/2024.01.26.577428; this version posted August 14, 2024. The copyright holder for this preprint (which was not certified by peer review) is the author/funder, who has granted bioRxiv a license to display the preprint in perpetuity. It is made available under aCC-BY 4.0 International license.

96 compounds at 10 µM for 30 minutes. Three compounds and a DMSO control were assayed in duplicate (batch). All 96 compounds and 64 DMSO controls were assayed using thirty-two treatment batches. Following treatment, an equal number of cells were transferred to 10 PCR tubes. The cells were placed in a thermal cycler and heated across a thermal gradient from 48°C-58°C for 3 minutes. The cells were allowed to cool to room temperature for 5 minutes. An equal volume from each PCR tube was pooled and spun at 300 x g for 3 minutes to pellet cells. Cells were washed one with PBS and lysed in a buffer containing 0.5% NP-40, which will dissolve membranes without disrupting heat-induced protein aggregates. The lysates were centrifuged for 90 minutes at 21,000 x g to separate soluble protein from aggregates. An equal volume of each soluble fraction (~20 µg) was prepared for LC-MS/MS analysis. Two treatment batches were combined for each TMTpro 16-plex. Each soluble fraction was reduced and alkylated. Each sample was precipitated onto SP3 carboxylate-coated beads to facilitate a buffer exchange. Proteomes were eluted off the SP3 beads into digestion buffer and digested with a combination of Lys-C and trypsin. Peptides from each sample were labeled with a unique TMTpro reagent. TMT-labelled peptides were pooled into a single sample, which was desalted using a sep-pak. Dried peptides were resuspended in HPLC buffer A and fractionated by basic reverse-phase HPLC. Twelve to twenty-four fractions were stage-tipped and analyzed on an Orbitrap Eclipse with a FAIMS device enabled (Thermo Fisher). Changes in thermal stability were determined by comparing soluble protein abundance in a compound-treated samples to vehicle-treated controls.

bioRxiv preprint doi: https://doi.org/10.1101/2024.01.26.577428; this version posted August 14, 2024. The copyright holder for this preprint (which was not certified by peer review) is the author/funder, who has granted bioRxiv a license to display the preprint in perpetuity. It is made available under aCC-BY 4.0 International license.

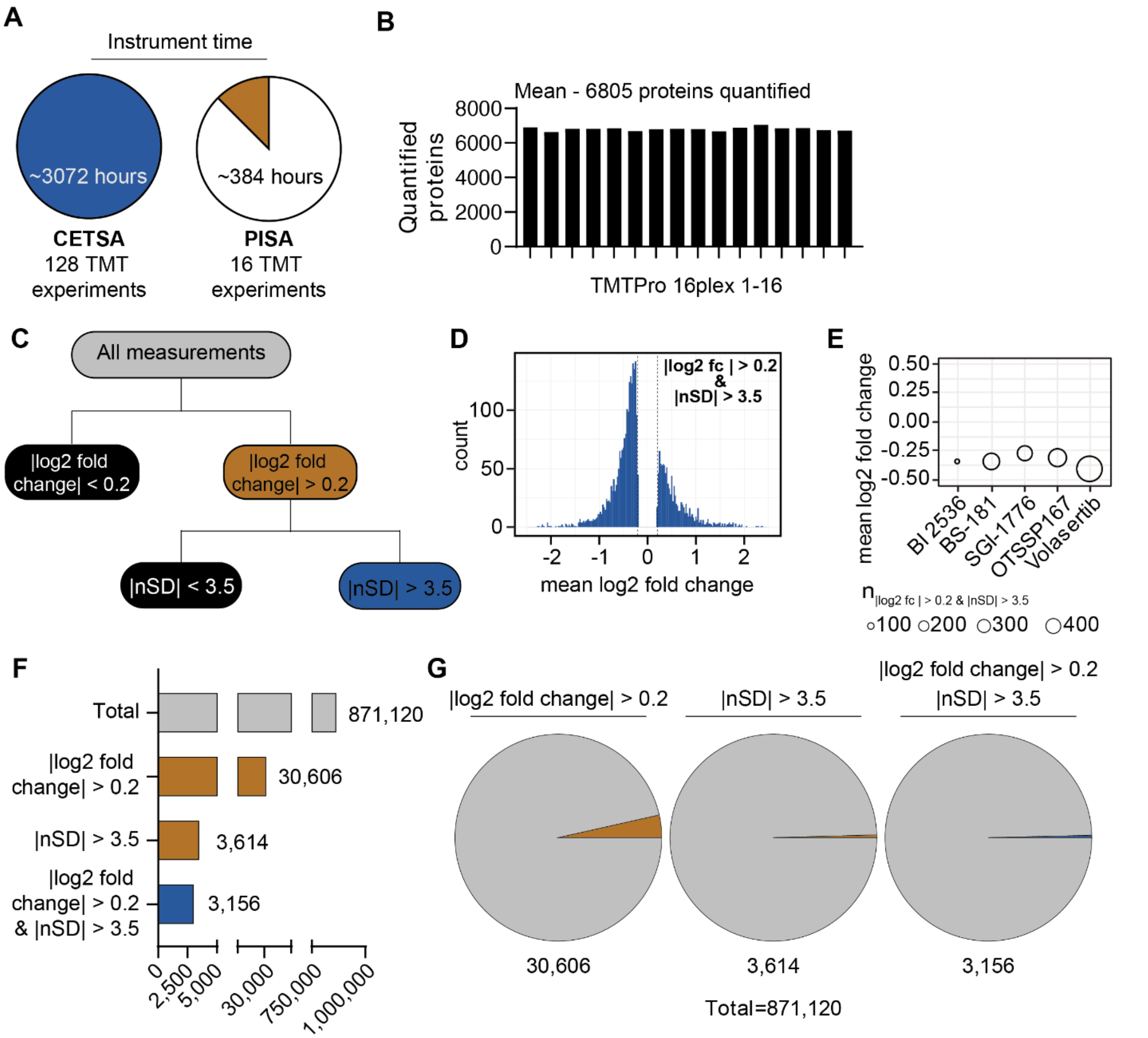

H

Protein	Compound	log2 fold change - Rep 1	log2 fold change - Rep 2	nSD - Rep 2	nSD - Rep 1
CDK1	AZD-5438	-0.1987977	-0.1995283	-5.0698165	-5.0884488
GRB2	Nilotinib	0.18163638	0.16685694	4.06035268	3.72996881
STK3	OTS167	-0.1593215	-0.1623354	-4.3108455	-4.3923962
AURKB	Tozasertib	1.00386214	1.21873672	2.63044095	3.1934813
GAK	CCT214533	1.45905736	1.41892828	3.25945758	3.165981132
GAK	Ralimetinib	1.10092618	1.15013176	2.45941131	2.569334

Figure 1 - Figure supplement 3

Figure 1 – figure supplement 3. Establishing a robust and efficient workflow for assessing changes in protein thermal stability in living cells. A. Minimal instrument time (assuming 12 fractions per plex) required to assay 96 compounds and 32 DMSO controls in duplicate using

bioRxiv preprint doi: https://doi.org/10.1101/2024.01.26.577428; this version posted August 14, 2024. The copyright holder for this preprint (which was not certified by peer review) is the author/funder, who has granted bioRxiv a license to display the preprint in perpetuity. It is made available under aCC-BY 4.0 International license.

CETSA (left) or PISA (right). B. Total proteins quantified per TMTpro 16-plex. C. Schematic depiction of the filtering scheme used to define significant changes. D. Histogram depicting the log_2 fold change measurements that were greater than 3.5 standard deviations from the mean. E,F. Total number of proteins passing each filter. G. Pie charts depicting the fraction of all measurements that pass each filter. H. Table highlighting proteins that pass the log_2 fold change filter, but not the nSD filter and vice versa.

bioRxiv preprint doi: https://doi.org/10.1101/2024.01.26.577428; this version posted August 14, 2024. The copyright holder for this preprint (which was not certified by peer review) is the author/funder, who has granted bioRxiv a license to display the preprint in perpetuity. It is made available under aCC-BY 4.0 International license.

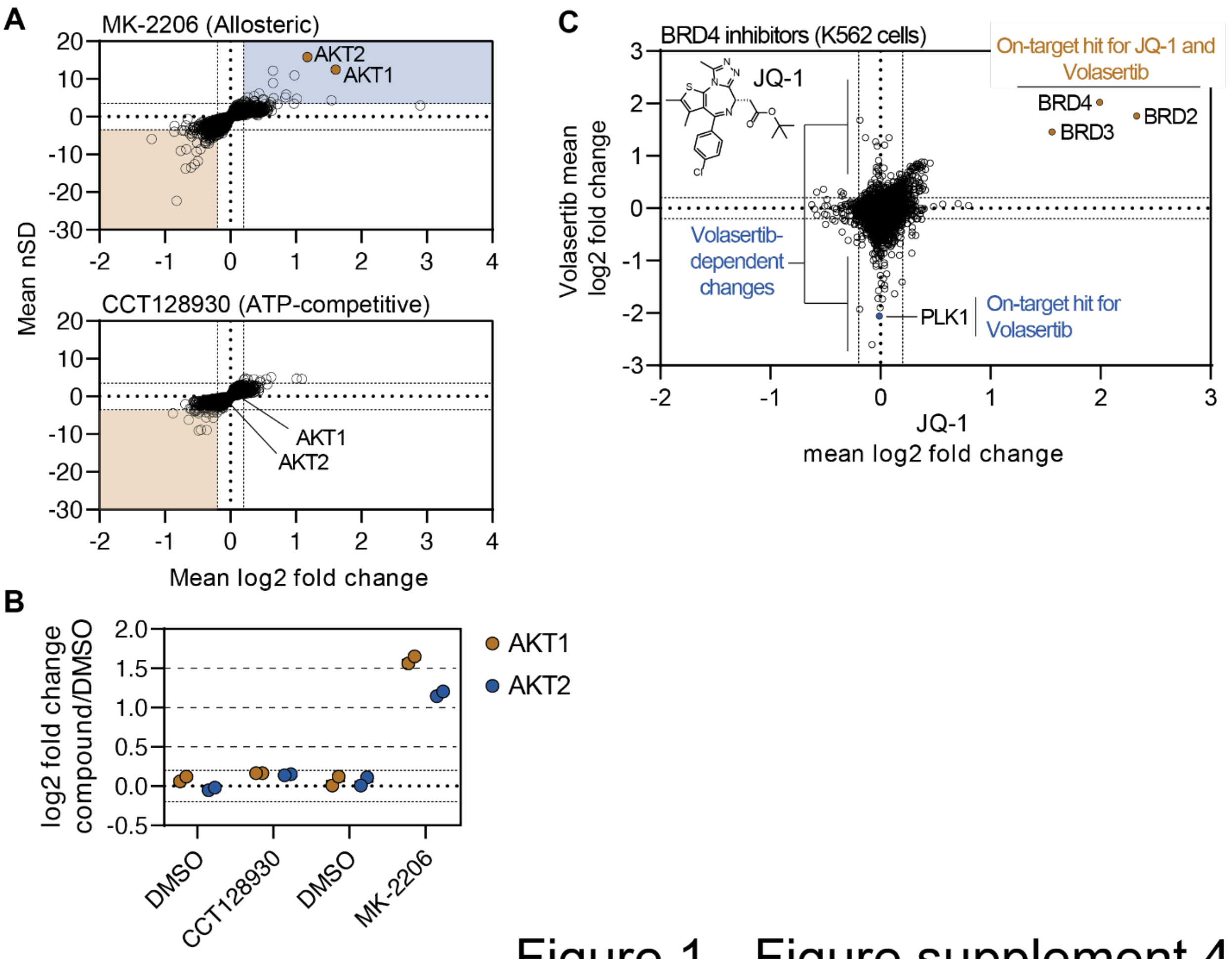

Figure 1 - Figure supplement 4

Figure 1 – figure supplement 4. Establishing a robust and efficient workflow for assessing changes in protein thermal stability in living cells. A-B. K562 cells (N=2) were treated with MK-2206 (top) or CCT128930 (bottom) at 10 µM for 30 minutes and assayed using PISA. The mean $\log_2$ fold change of duplicate measurements is plotted on the x-axis and the mean nSD is plotted on the y-axis. Blue boxes contain proteins that exhibit an increase in solubility (increase in melting temperature) and orange boxes contain proteins that exhibit a decrease in solubility (decrease in melting temperature). Orange points represent the known target of each compound. B. The plot displays the $\log_2$ fold change values for selected proteins following treatment with each compound in A. C. Plot comparing the $\log_2$ fold changes measurements for each protein in JQ-1 (x-axis)- and volasertib (y-axis)-treated K562 cells. Orange points represent the known targets of the compounds.

bioRxiv preprint doi: https://doi.org/10.1101/2024.01.26.577428; this version posted August 14, 2024. The copyright holder for this preprint (which was not certified by peer review) is the author/funder, who has granted bioRxiv a license to display the preprint in perpetuity. It is made available under aCC-BY 4.0 International license.

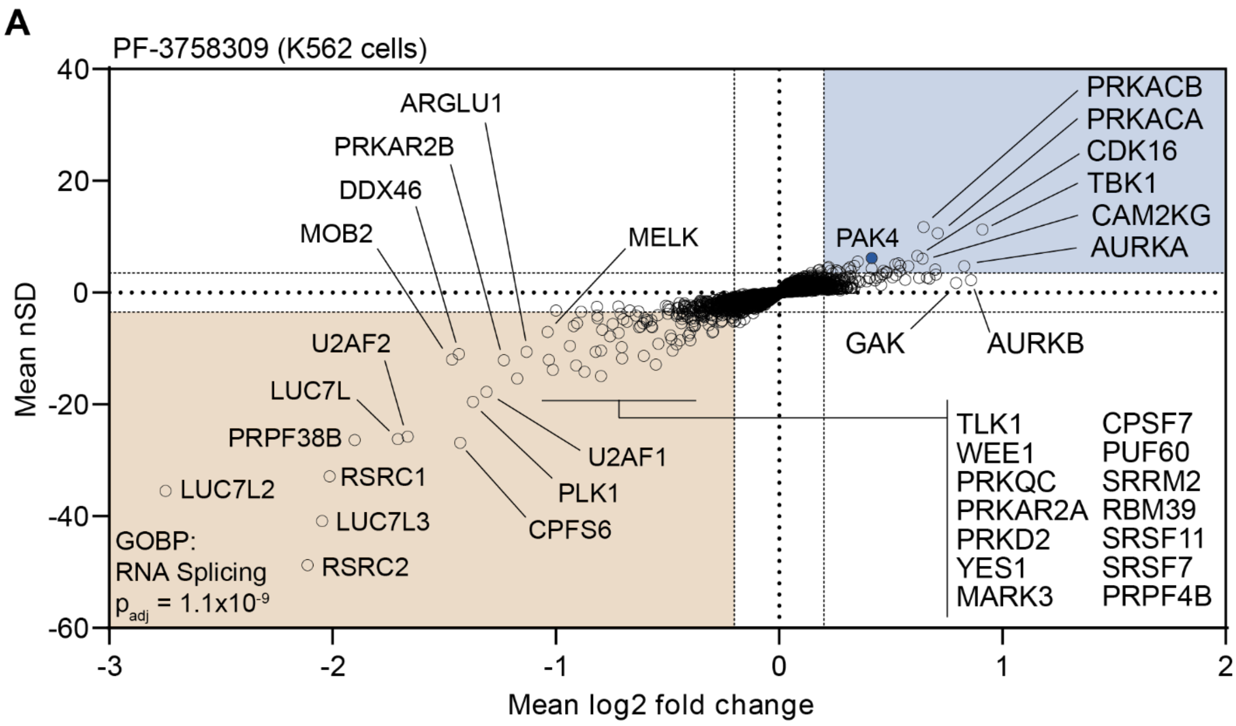

Figure 1 - Figure supplement 5

Figure 1 – figure supplement 5. Establishing a robust and efficient workflow for assessing changes in protein thermal stability in living cells. A. Plot highlighting the significant changes in protein thermal stability following treatment with PF-3758309. The mean log_2 fold change of duplicate measurements is plotted on the x-axis and the mean nSD is plotted on the y-axis. Blue boxes contain proteins that exhibit an increase in solubility (increase in melting temperature) and orange boxes contain proteins that exhibit a decrease in solubility (decrease in melting temperature). The blue point represents the known target.

bioRxiv preprint doi: https://doi.org/10.1101/2024.01.26.577428; this version posted August 14, 2024. The copyright holder for this preprint (which was not certified by peer review) is the author/funder, who has granted bioRxiv a license to display the preprint in perpetuity. It is made available under aCC-BY 4.0 International license.

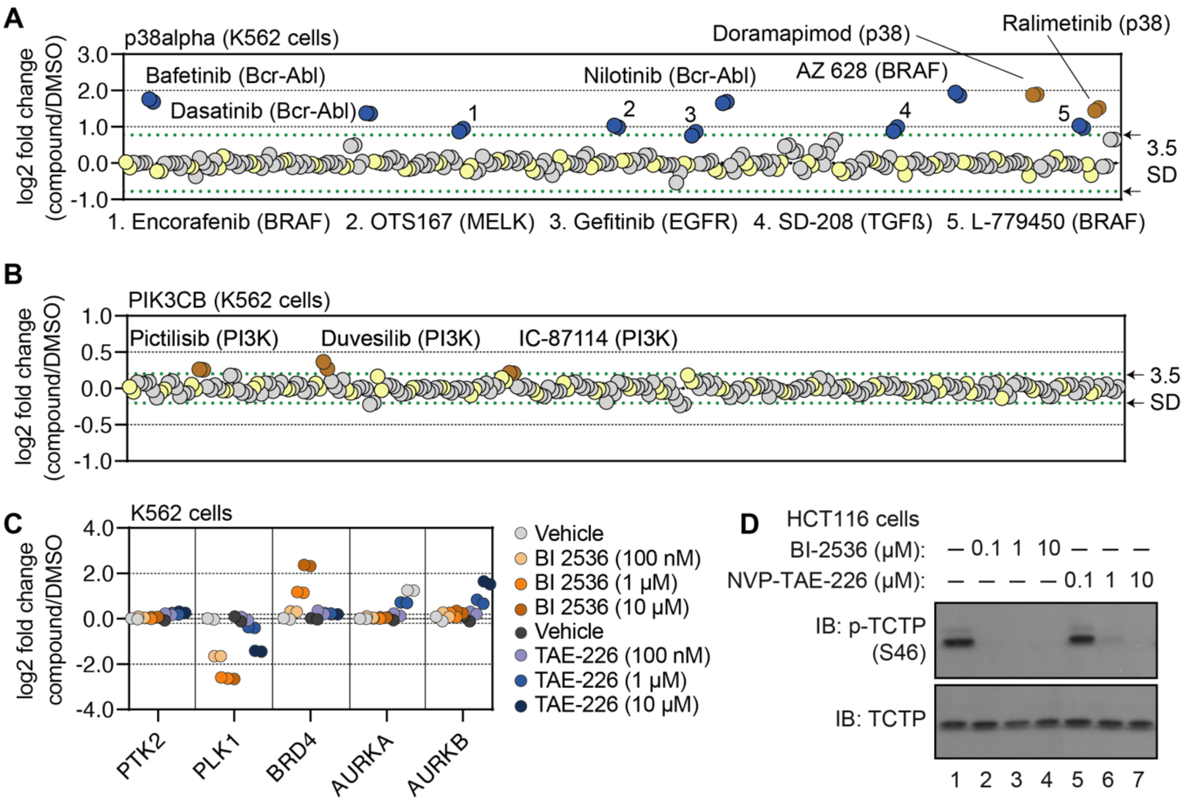

Figure 2 - Figure supplement 1

Figure 2 – figure supplement 1. Using cell-based PISA data to assess compound target engagement. A-B. A protein-centric view of p38α (A) and PIK3CB (B) solubility (thermal stability) in response to all treatments. Log_2 fold change is plotted on the y-axis. The points represent each of the 256 treatments that were performed. Orange points indicate compounds known to target each respective protein. Blue points indicate other compounds that result in a significant thermal stabilization. Green dashed lines mark a SD of 3.5 cutoff for each treatment. C-D. K562 cells were treated with the indicated concentrations of NVP-TAE-226 or BI 2536 for 15 minutes. Changes in protein thermal stability are represented as a log_2 fold change in soluble protein abundance for each treatment in reference to a DMSO-treated control (C). PLK1 activity was determined using a western blot for p-TCTP (S46) and total TCTP (D).

bioRxiv preprint doi: https://doi.org/10.1101/2024.01.26.577428; this version posted August 14, 2024. The copyright holder for this preprint (which was not certified by peer review) is the author/funder, who has granted bioRxiv a license to display the preprint in perpetuity. It is made available under aCC-BY 4.0 International license.

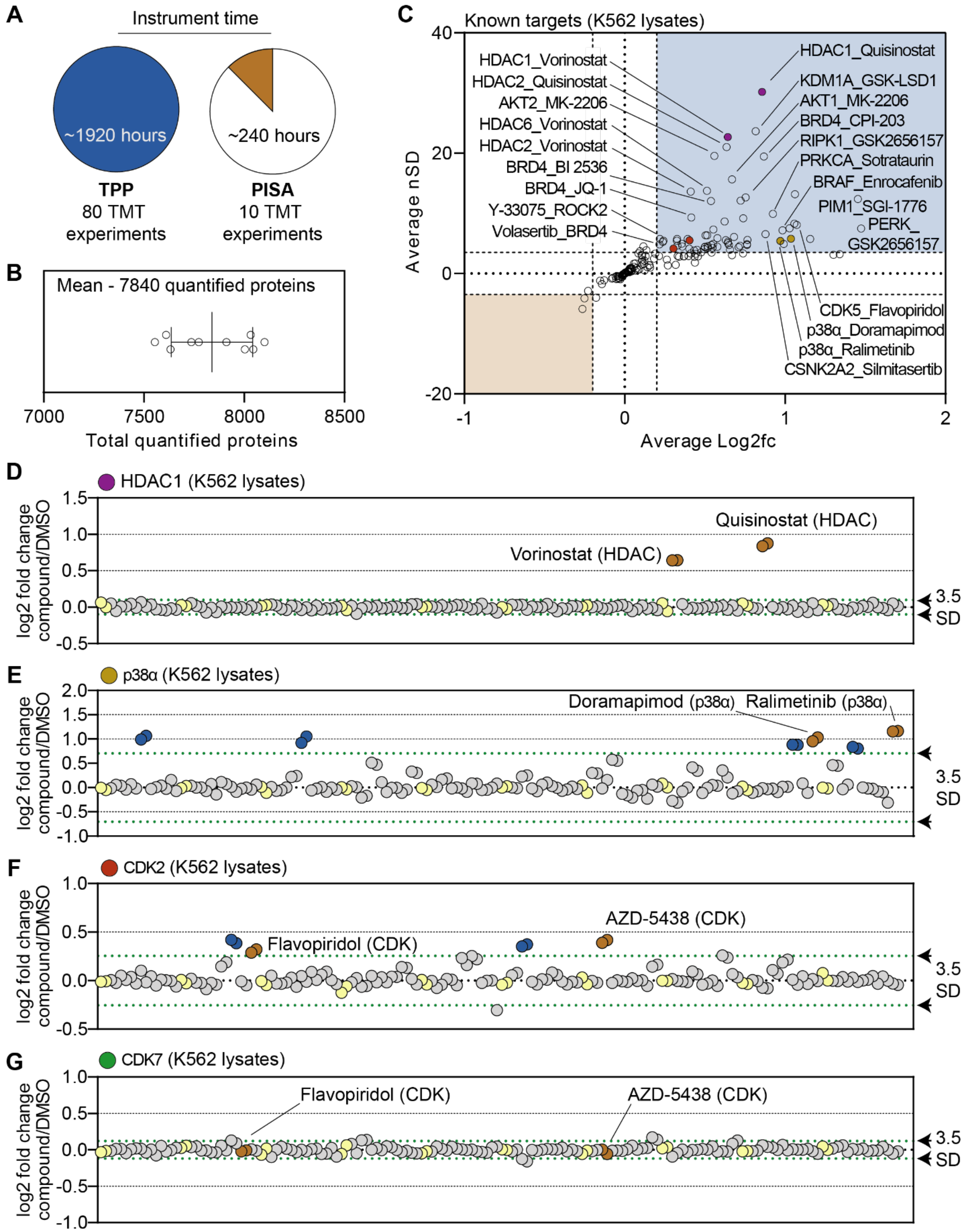

Figure 3 - Figure supplement 1

1

bioRxiv preprint doi: https://doi.org/10.1101/2024.01.26.577428; this version posted August 14, 2024. The copyright holder for this preprint (which was not certified by peer review) is the author/funder, who has granted bioRxiv a license to display the preprint in perpetuity. It is made available under aCC-BY 4.0 International license.

Figure 3 – figure supplement 1. A chemical perturbation screen of 70 compounds in K562 native extracts. A. Minimal instrument time required to assay 70 compounds and 10 DMSO controls in duplicate using TPP (left) or PISA (right). B. Total number of proteins quantified in each plex in the lysate-based screen. C. A plot of the mean $\log_2$ fold change (x-axis) and mean nSD (y-axis) of each known compound-target pair that were quantified in the lysate-based screen. Blue boxes contain proteins that exhibit an increase in solubility (increase in melting temperature) and orange boxes contain proteins that exhibit a decrease in solubility (decrease in melting temperature). D-G. Protein-centric view of HDAC1 (D), p38α (E), CDK2 (F), and CDK7 (G) solubility (thermal stability) in response to all treatments. Orange points indicate compounds known to target each respective protein. Blue points indicate other compounds that result in a significant thermal destabilization of each respective protein. Green dashed lines mark a SD of 3.5 cutoff for each compound.

bioRxiv preprint doi: https://doi.org/10.1101/2024.01.26.577428; this version posted August 14, 2024. The copyright holder for this preprint (which was not certified by peer review) is the author/funder, who has granted bioRxiv a license to display the preprint in perpetuity. It is made available under aCC-BY 4.0 International license.

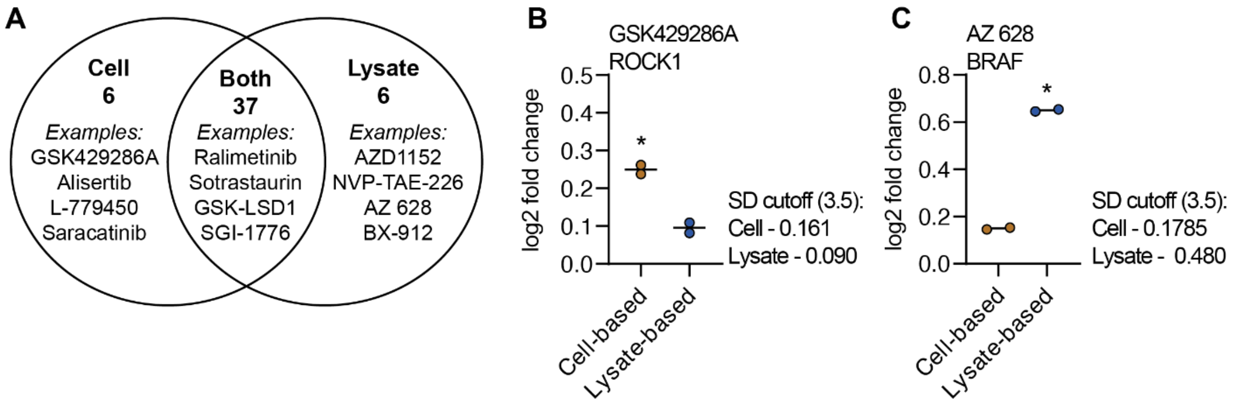

Figure 3 - Figure supplement 2

Figure 3 – figure supplement 2. A chemical perturbation screen of 70 compounds in K562 native extracts. A. A venn diagram depicting the total number of compounds that cause a change in a known target in cell- and lysate-based PISA. B,C. Log2 fold change measurements for the indicated drug-target pair (GSK429286A_ROCK1 [A] and AZ 628_BRAF [B]) in cell- and lysate-based PISA.

bioRxiv preprint doi: https://doi.org/10.1101/2024.01.26.577428; this version posted August 14, 2024. The copyright holder for this preprint (which was not certified by peer review) is the author/funder, who has granted bioRxiv a license to display the preprint in perpetuity. It is made available under aCC-BY 4.0 International license.

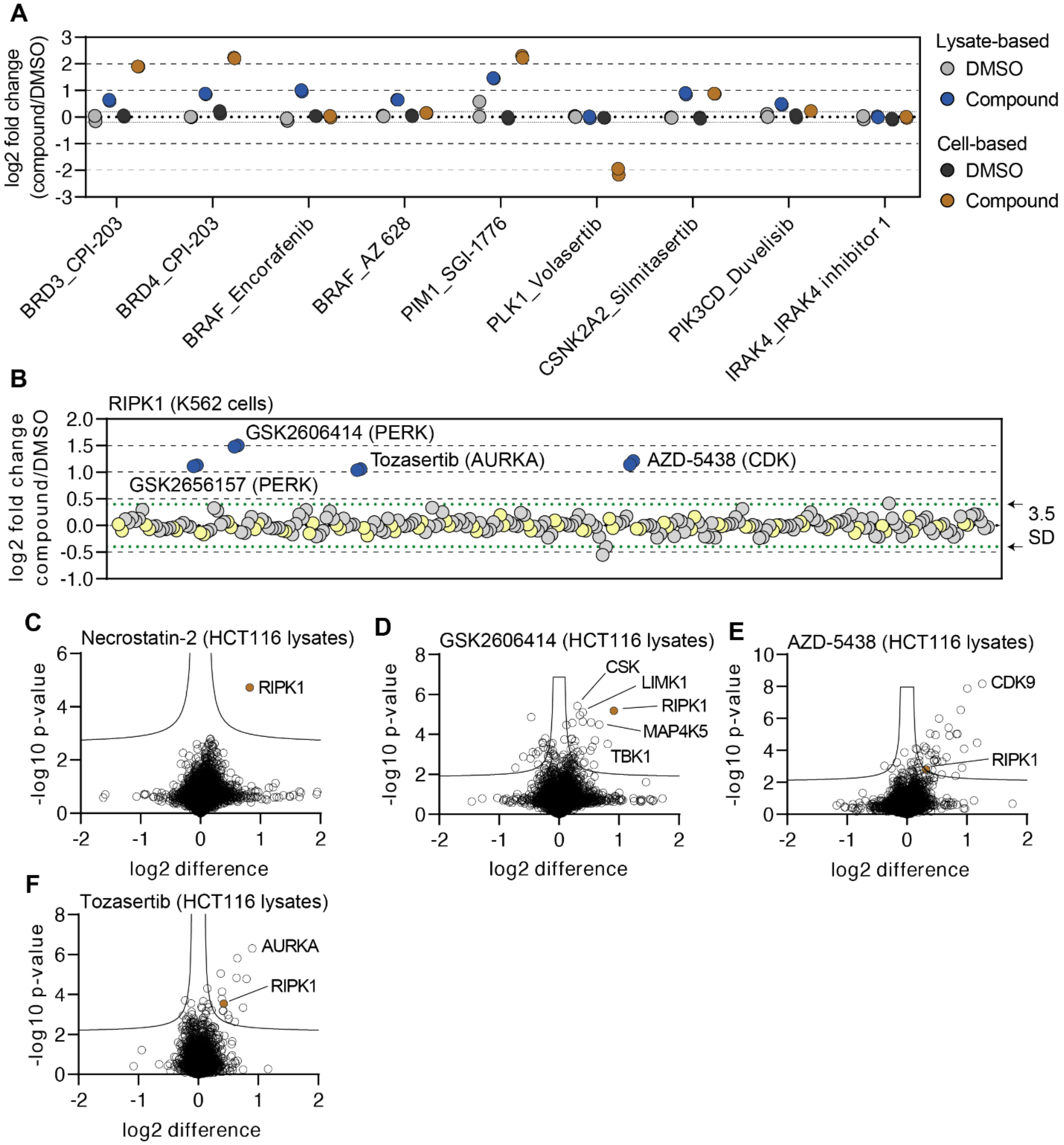

Figure 4 - Figure supplement 1

Figure 4 – figure supplement 1. Cell- and lysate-based PISA are complimentary approaches for assessing mechanism of action. A. Changes in protein solubility (thermal stability) for the indicated proteins in cell- and lysate-based PISA are represented as a log_2 fold change in protein abundance for each treatment in reference to a DMSO-treated control. Dashed lines indicate a log_2 fold change of +/-0.2. B. A protein-centric view of RIPK1 solubility (thermal stability) in response to all treatments. Log_2 fold change is plotted on the y-axis. The points represent each of the 256 treatments that were performed. Blue points highlight compounds that result in a significant increase in solubility (thermal stabilization). Green dashed lines mark a SD of 3.5 cutoff

bioRxiv preprint doi: https://doi.org/10.1101/2024.01.26.577428; this version posted August 14, 2024. The copyright holder for this preprint (which was not certified by peer review) is the author/funder, who has granted bioRxiv a license to display the preprint in perpetuity. It is made available under aCC-BY 4.0 International license.

for RIPK1. C-F. HCT116 lysates were treated with necrostatin-2 (C), GSK2606414 (D), AZD-5438 (E), or tozasertib (F) (N=3) or DMSO (N=4) for 15 minutes and any changes in thermal stability were determined using PISA. Data is presented as a volcano plot to highlight significant changes in abundance. Significant changes were determined using a permutation-based FDR (FDR – 0.05, S0 – 0.1).

bioRxiv preprint doi: https://doi.org/10.1101/2024.01.26.577428; this version posted August 14, 2024. The copyright holder for this preprint (which was not certified by peer review) is the author/funder, who has granted bioRxiv a license to display the preprint in perpetuity. It is made available under aCC-BY 4.0 International license.

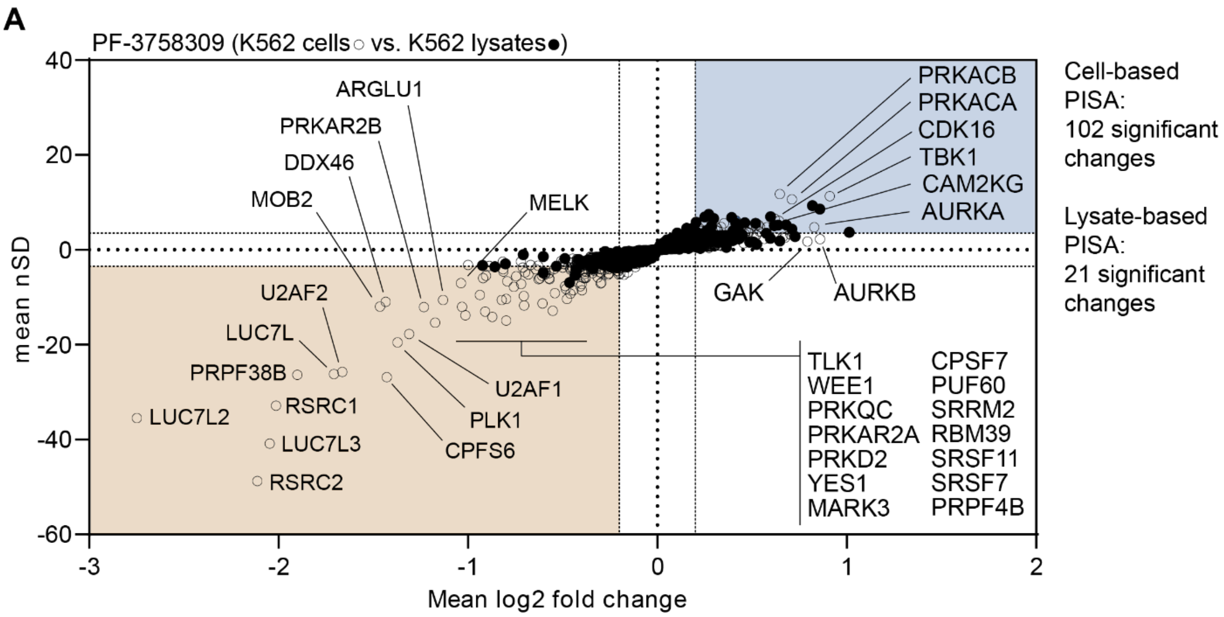

Figure 5 - Figure supplement 1

Figure 5 – figure supplement 1. Disparities in cell- and lysate-based PISA can point toward secondary changes in protein thermal stability. A. Plot highlighting the significant changes in protein solubility (thermal stability) following treatment with PF-3758309 in cells (no fill) and lysates (black points). The mean $\log_2$ fold change of duplicate measurements is plotted on the x-axis and the mean nSD is plotted on the y-axis. Blue boxes contain proteins that exhibit an increase in solubility (increase in melting temperature) and orange boxes contain proteins that exhibit a decrease in solubility (decrease in melting temperature).

bioRxiv preprint doi: https://doi.org/10.1101/2024.01.26.577428; this version posted August 14, 2024. The copyright holder for this preprint (which was not certified by peer review) is the author/funder, who has granted bioRxiv a license to display the preprint in perpetuity. It is made available under aCC-BY 4.0 International license.

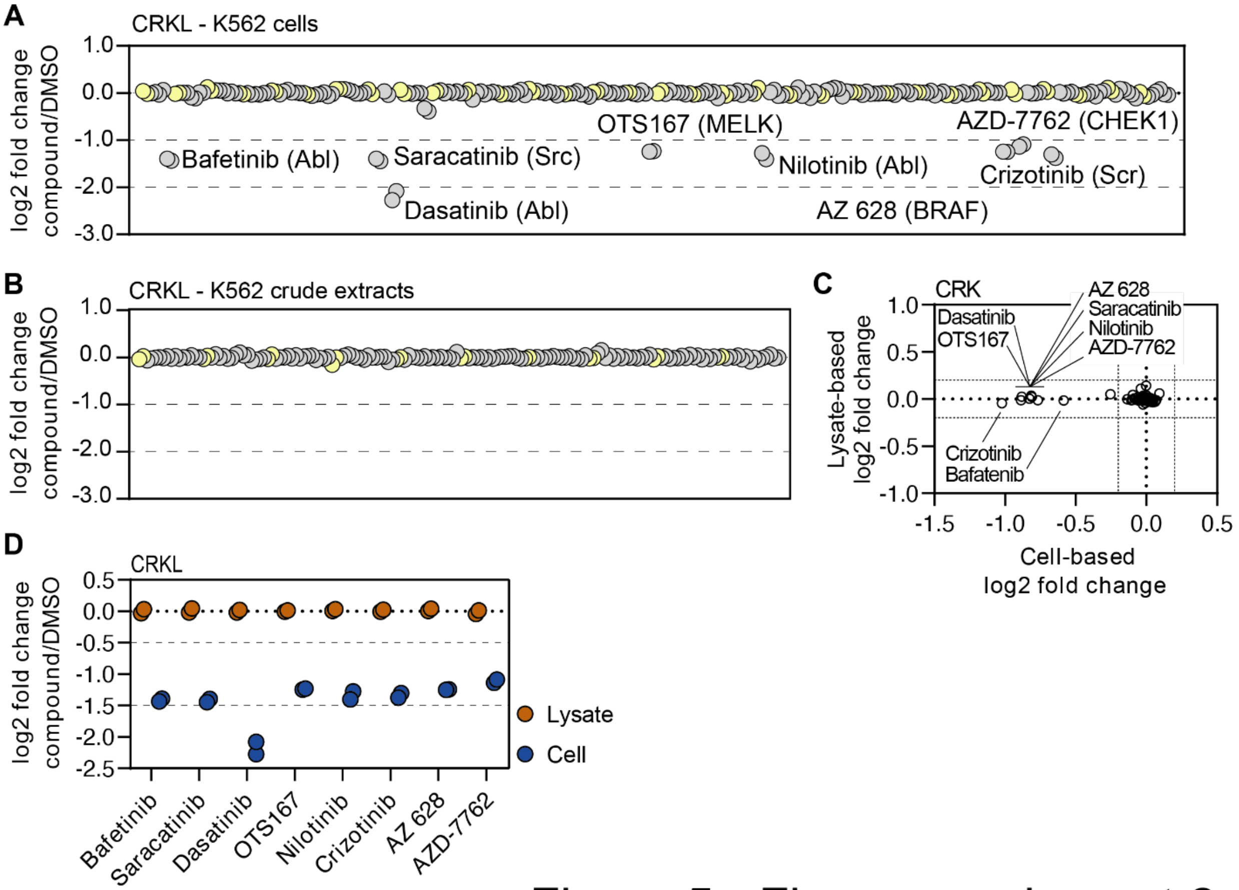

Figure 5 - Figure supplement 2

Figure 5 – figure supplement 2. Disparities in cell- and lysate-based PISA can point toward secondary changes in protein thermal stability. A and B. A protein-centric view of CRKL solubility (thermal stability) in K562 cells (A) and lysates (B) in response to all treatments. Log_2 fold change is plotted on the y-axis. The points represent each treatment that was performed in each screen. Green dashed lines mark a SD of 3.5 cutoff for each treatment. C. Plots depicting the log_2 fold change values for CRK in response to treatment with all compounds assayed using cell- and lysate-based PISA. D. Changes in protein solubility (thermal stability) for the indicated proteins in cell- and lysate-based PISA are represented as a log_2 fold change in soluble protein abundance for each treatment in reference to a DMSO-treated control.

bioRxiv preprint doi: https://doi.org/10.1101/2024.01.26.577428; this version posted August 14, 2024. The copyright holder for this preprint (which was not certified by peer review) is the author/funder, who has granted bioRxiv a license to display the preprint in perpetuity. It is made available under aCC-BY 4.0 International license.

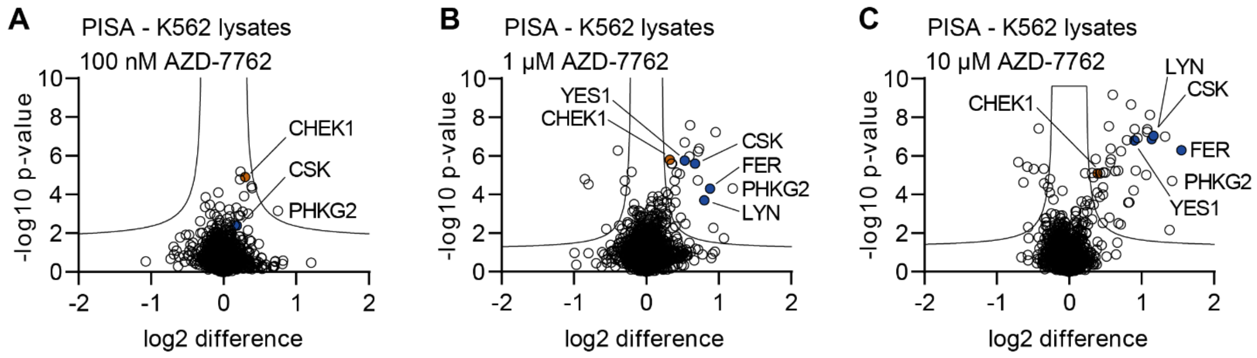

Figure 5 - Figure supplement 3

Figure 5 – figure supplement 3. Disparities in cell- and lysate-based PISA can point toward secondary changes in protein thermal stability. A-C. K562 lysates were treated with the indicated concentrations of AZD-7762 for 15 minutes and assayed using PISA. Changes in protein thermal stability are represented as a $\log_2$ fold change in protein soluble abundance for each treatment in reference to a DMSO-treated control. Significant changes were determined using a permutation-based FDR (FDR – 0.05, S0 – 0.1).

bioRxiv preprint doi: https://doi.org/10.1101/2024.01.26.577428; this version posted August 14, 2024. The copyright holder for this preprint (which was not certified by peer review) is the author/funder, who has granted bioRxiv a license to display the preprint in perpetuity. It is made available under aCC-BY 4.0 International license.

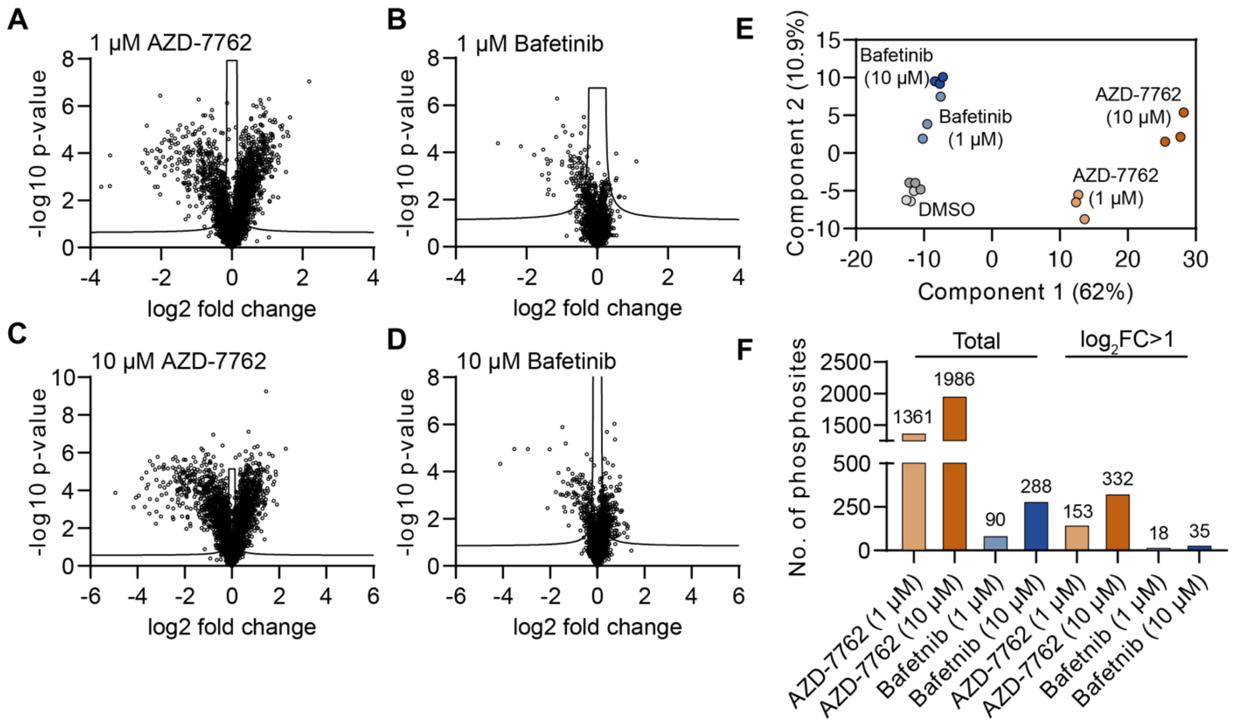

Figure 5 - Figure supplement 4

Figure 5 – figure supplement 4. Disparities in cell- and lysate-based PISA can point toward secondary changes in protein thermal stability. A-F. K562 cells were treated with the indicated concentration of bafetinib or AZD-7762 for 15 minutes and assayed using phosphoproteomic profiling. A-D. Data is displayed as a volcano plot. Significant changes were determined using a permutation-based FDR (FDR – 0.05, S0 – 0.1). E. PCA plot of phosphoproteomic data. F. Total number of significant changes that result from each treatment (left) or significant changes with a log2 fold change > 1 or < -1 (right).

bioRxiv preprint doi: https://doi.org/10.1101/2024.01.26.577428; this version posted August 14, 2024. The copyright holder for this preprint (which was not certified by peer review) is the author/funder, who has granted bioRxiv a license to display the preprint in perpetuity. It is made available under aCC-BY 4.0 International license.

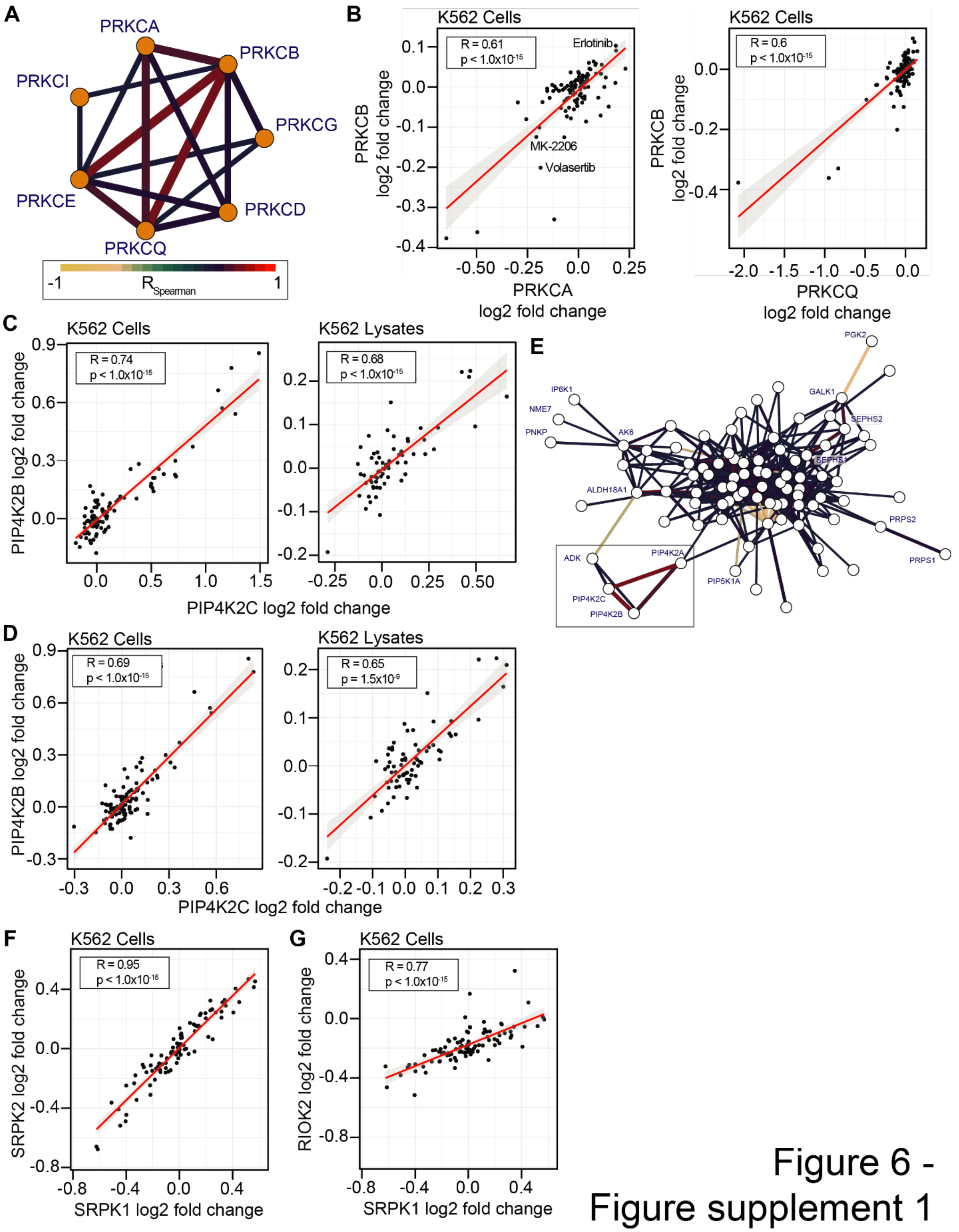

Figure 6 – figure supplement 1. Concordance of protein engagement for protein complex members and structurally similar kinases. A. Subgraph from Figure 7A highlighting the

bioRxiv preprint doi: https://doi.org/10.1101/2024.01.26.577428; this version posted August 14, 2024. The copyright holder for this preprint (which was not certified by peer review) is the author/funder, who has granted bioRxiv a license to display the preprint in perpetuity. It is made available under aCC-BY 4.0 International license.

correlation between PRKC kinases. Edges are colored based on $R_{Spearman}$. B. Correlation of PRKCB and either PRKCA or PRKCQ. These correlations are driven by consistent but relatively small changes in thermal stability. C. Non-protein kinases of the correlation network graph. Library compounds did not specifically target any non-protein kinases though these compounds elicited consistent solubility (thermal stability) changes in these kinases. Comparison of compound engagement for PIP4K2 non-protein kinases (D) PIP4K2A and PIP4K2B as well as (E) PIP4K2B and PIP4K2C. Compound engagement generates statistically significant correlations in both cells and lysates, with a larger solubility (thermal stability) effect size in cell-based assays. F-G. Significant correlation with small solubility (thermal stability) fold changes for the non-protein kinases SRPK1, SRPK2, and RIOK2.

bioRxiv preprint doi: https://doi.org/10.1101/2024.01.26.577428; this version posted August 14, 2024. The copyright holder for this preprint (which was not certified by peer review) is the author/funder, who has granted bioRxiv a license to display the preprint in perpetuity. It is made available under aCC-BY 4.0 International license.

Table 1. Library compounds. A list of all compounds utilized in the cell- and lysate-based PISA screens.

Table 2. Sample multiplexing layout. This table contains the TMTPro channel assignments for all samples generated in the cell- and lysate-based screens.

Table 3. Cell-based PISA full dataset. This table contains all the data from the cell-based screen in an easy to graph format.

Table 4. Lysate-based PISA full dataset. This table contains all the data from the lysate-based screen in an easy to graph format.

Figure 1 – source data 1. This table contains cell-based PISA data for K562 cells treated with ribociclib, palbociclib, and abemaciclib.

Figure 1 – source data 2. This table contains the PISA data for cell- and lysate-based screens.

Figure 2 – source data 1. This table contains cell-based PISA data for K562 cells treated with increasing concentrations of BI 2536 and palbocilicb.

Figure 2 – source data 2. This table contains cell-based PISA data for K562 cells treated with increasing concentrations of BI 2536 and NVP-TAE-226.

Figure 2 – source data 3. This document contains the unedited western scans for the panels displayed in Figure 2F and Figure 2 – figure supplement 1D.

Figure 4 – source data 1. This table contains lysate-based PISA data for HCT116 lysates treated with necrostatin-2, GSK2606414, AZD-5438, and tozasertib.

Figure 4 – source data 2. This document contains the unedited western scans for the panel displayed in Figure 4G.

Figure 5 – source data 1. This document contains the unedited western scans for the panels displayed in Figure 5C.

Figure 5 – source data 2. This table contains lysate-based PISA data for K562 lysates treated with 100 nM, 1 μM, or 10 μM AZD-7762.

Figure 5 – source data 3. This table contains phosphoproteomic data for K562 cells treated with 1 μM AZD-7762, 10 μM AZD-7762, 1 μM bafetinib, or 10 μM bafetinib.

Figure 5 – source data 4. This table contains proteomic data for K562 cells treated with 1 μM AZD-7762, 10 μM AZD-7762, 1 μM bafetinib, or 10 μM bafetinib.

END
