# Supplementary material for: Large-scale characterization of drug mechanism of action using proteome-wide thermal shift assays
Source: bioRxiv. 2024 Feb 10:2024.01.26.577428. Originally published 2024 Jan 27. Preprint. [Version 3] doi: 10.1101/2024.01.26.577428 (PMC10849652; doi:10.1101/2024.01.26.577428)
Supplement: Supplementary file 2 [file media-11.pdf]

Key Resource Table

Reagent type	Designation	Source or reference	Identifier
Antibody	anti-CRKL (rabbit monoclonal antibody)	Cell Signaling Technologies	38710
Antibody	anti-p-CRKL Y207 (rabbit monoclonal antibody)	Cell Signaling Technologies	3490
Antibody	anti-RIPK1 (rabbit monoclonal antibody)	Cell Signaling Technologies	3493
Antibody	anti-p-RIPK1 S166 (rabbit monoclonal antibody)	Cell Signaling Technologies	65746
Antibody	anti-TCTP (rabbit monoclonal antibody)	Cell Signaling Technologies	5128
Antibody	anti-p-TCTP (rabbit monoclonal antibody)	Cell Signaling Technologies	5251
Antibody	goat anti-rabbit IgG-HRP secondary antibody	Santa Cruz Biotechnology	sc-2004
Cell line (*H. sapiens*)	K562	ATCC	
Cell line (*H. sapiens*)	HCT116	ATCC	
Chemical compound	AZD1152	MedChemExpress	HY-10127
Chemical compound	Rosiglitazone	MedChemExpress	HY-17386
Chemical compound	Bafetinib	MedChemExpress	HY-50868
Chemical compound	AS-605240	MedChemExpress	HY-10109
Chemical compound	MP7	MedChemExpress	HY-14440
Chemical compound	Ibrutinib	MedChemExpress	HY-10997
Chemical compound	Gefitinib	MedChemExpress	HY-50895
Chemical compound	AZD-5438	MedChemExpress	HY-10012
Chemical compound	Nilotinib	MedChemExpress	HY-10159
Chemical compound	GSK429286A	MedChemExpress	HY-11000
Chemical compound	PF-3758309	MedChemExpress	HY-13007
Chemical compound	GSK126	MedChemExpress	HY-13470
Chemical compound	OSI-027	MedChemExpress	HY-10423
Chemical compound	Mubritinib	MedChemExpress	HY-13501

Key Resource Table

Chemical compound	Alisertib	MedChemExpress	HY-10971
Chemical compound	MK-2206 (dihydrochloride)	MedChemExpress	HY-10358
Chemical compound	Silmitasertib	MedChemExpress	HY-50855
Chemical compound	GSK343	MedChemExpress	HY-13500
Chemical compound	NVP-TAE 226	MedChemExpress	HY-13203
Chemical compound	BS-181	MedChemExpress	HY-13266
Chemical compound	Erlotinib	MedChemExpress	HY-50896
Chemical compound	BML-277	MedChemExpress	HY-13946
Chemical compound	BMS-911543	MedChemExpress	HY-15270
Chemical compound	Givinostat	Cayman	11045
Chemical compound	LY2090314	MedChemExpress	HY-16294
Chemical compound	SD-208	MedChemExpress	HY-13227
Chemical compound	AZ20	MedChemExpress	HY-15557
Chemical compound	Epoxomicin	MedChemExpress	HY-13821
Chemical compound	Nexturastat A	MedChemExpress	HY-16699
Chemical compound	TAK-285	MedChemExpress	HY-15196
Chemical compound	Sotrastaurin	MedChemExpress	HY-10343
Chemical compound	AZ 628	MedChemExpress	HY-11004
Chemical compound	VE-821	MedChemExpress	HY-14731
Chemical compound	Entinostat	MedChemExpress	HY-12163
Chemical compound	Filanesib	MedChemExpress	HY-15187
Chemical compound	AZD-7762	MedChemExpress	HY-10992
Chemical compound	Crizotinib	MedChemExpress	HY-50878
Chemical compound	PCI-34051	MedChemExpress	HY-15224
Chemical compound	Pinometostat	MedChemExpress	HY-15593

Key Resource Table

Chemical compound	SB 525334	MedChemExpress	HY-12043
Chemical compound	IRAK-1-4 Inhibitor I	MedChemExpress	HY-13329
Chemical compound	Doramapimod	MedChemExpress	HY-10320
Chemical compound	JNJ-38877605	MedChemExpress	HY-50683
Chemical compound	ZM 336372	MedChemExpress	HY-13343
Chemical compound	Ispinesib	MedChemExpress	HY-50759
Chemical compound	MG-132	MedChemExpress	HY-13259
Chemical compound	L-779450	MedChemExpress	HY-12787
Chemical compound	Ralimetinib	Cayman	23259
Chemical compound	Pictilisib	MedChemExpress	HY-50094
Chemical compound	GSK2656157	MedChemExpress	HY-13820
Chemical compound	GSK-1070916	MedChemExpress	HY-70044
Chemical compound	SGI-1776	MedChemExpress	HY-13287
Chemical compound	Gandotinib	MedChemExpress	HY-13034
Chemical compound	Vorinostat	MedChemExpress	HY-10221
Chemical compound	GSK2606414	MedChemExpress	HY-18072
Chemical compound	Y-33075 (dihydrochloride)	MedChemExpress	HY-10069
Chemical compound	CPI-203	MedChemExpress	HY-15846
Chemical compound	Zotarolimus	MedChemExpress	HY-12424
Chemical compound	GSK-LSD1 (dihydrochloride)	MedChemExpress	HY-100546A
Chemical compound	Flavopiridol	MedChemExpress	HY-10005
Chemical compound	Duvelisib	MedChemExpress	HY-17044
Chemical compound	TEPP-46	MedChemExpress	HY-18657
Chemical compound	Saracatinib	MedChemExpress	HY-10234
Chemical compound	Dasatinib	MedChemExpress	HY-10181

Key Resource Table

Chemical compound	GW 501516	MedChemExpress	HY-10838
Chemical compound	GSK2334470	MedChemExpress	HY-14981
Chemical compound	CCT128930	MedChemExpress	HY-13260
Chemical compound	Tozasertib	MedChemExpress	HY-10161
Chemical compound	BX-912	MedChemExpress	HY-11005
Chemical compound	(+)-JQ-1	MedChemExpress	HY-13030
Chemical compound	Tubastatin A	MedChemExpress	HY-13271A
Chemical compound	ER-27319 maleate	Tocris	2471
Chemical compound	BI 2536	MedChemExpress	HY-50698
Chemical compound	CC-401 (hydrochloride)	MedChemExpress	HY-13022
Chemical compound	Encorafenib	MedChemExpress	HY-15605
Chemical compound	Entospletinib	MedChemExpress	HY-15968
Chemical compound	Rucaparib (Camsylate)	MedChemExpress	HY-102003
Chemical compound	Citarinostat	Cayman	26173
Chemical compound	IC-87114	MedChemExpress	HY-10110
Chemical compound	Vemurafenib	MedChemExpress	HY-12057
Chemical compound	OSU-03012	MedChemExpress	HY-10547
Chemical compound	Defactinib	MedChemExpress	HY-12289
Chemical compound	PJ34 (hydrochloride)	MedChemExpress	HY-13688
Chemical compound	FTI-277 (hydrochloride)	MedChemExpress	HY-15872A
Chemical compound	Idasanutlin	MedChemExpress	HY-15676
Chemical compound	SP600125	MedChemExpress	HY-12041
Chemical compound	PRT062607 (Hydrochloride)	MedChemExpress	HY-15323
Chemical compound	Ro-3306	MedChemExpress	HY-12529
Chemical compound	OTSSP167 (hydrochloride)	MedChemExpress	HY-15512A

Key Resource Table

Chemical compound	SP2509	MedChemExpress	HY-12635
Chemical compound	Lapatinib	MedChemExpress	HY-50898
Chemical compound	PLX-4720	MedChemExpress	HY-51424
Chemical compound	CCT241533 (hydrochloride)	MedChemExpress	HY-14715B
Chemical compound	Volasertib	MedChemExpress	HY-12137
Chemical compound	Tipifarnib	MedChemExpress	HY-10502
Chemical compound	A205804	Cayman	21252
Chemical compound	Quisinostat	MedChemExpress	HY-15433
Chemical compound	Palbociclib	Cayman	12673
Chemical compound	Abemaciclib	Cayman	21560
Chemical compound	Ribociclib	Cayman	17666
Chemical compound	BI 2536	Cayman	17385
Chemical compound	NVP-TAE 226	Cayman	17684
Chemical compound	Necrostatin-2	Cayman	11657
Chemical compound	GSK2606414	Cayman	17367
Chemical compound	AZD-5438	Cayman	21598
Chemical compound	Tozaserib	SYNKinase	SYN-1092-M001
Chemical compound	Bafetinib	Cayman	19169
Chemical compound	AZD7762	Cayman	11491
Chemical compound	Nocodazole	Cayman	13857
Chemical compound	Z-VAD(Ome)-FMK	Cayman	14463
Chemical compound	Birinapant	Cayman	19699
Recombinant protein	Human recombinant TNF-α	Cayman	32020
Software	Prism	GraphPad	10.0.0
Software	Perseus	maxquant.net/perseus	

Key Resource Table

Software	R	https://www.r-project.org/	
